# Complete Genome Sequence of Shigella sonnei Strain SE6-1, Capable of Selenate Reduction

**DOI:** 10.1128/MRA.00135-21

**Published:** 2021-04-01

**Authors:** Kathyleen Nogrado, Ahyeon Cho, Dukki Han, Cuong Tu Ho, James K. Fredrickson, Ji-Hoon Lee

**Affiliations:** a Department of Bioenvironmental Chemistry, Jeonbuk National University, Jeonju, Jeollabuk-do, Republic of Korea; b Department of Marine Molecular Bioscience, Gangneung-Wonju National University, Gangneung, Ganwon-do, Republic of Korea; c Institute of Environmental Technology, Hanoi, Vietnam; d Pacific Northwest National Laboratory, Richland, Washington, USA; Portland State University

## Abstract

We report the complete genome sequence of selenate [Se(VI)]-reducing Shigella sonnei SE6-1, which was isolated from stream sediment from an industrial complex in Jeonju, South Korea. The genome sequence is 4,762,774 bp long, with a G+C content of 50.7% and 4,548 genes, including 4,440 coding sequences, 22 rRNA genes, and 86 tRNA genes.

## ANNOUNCEMENT

**FIG 1:**
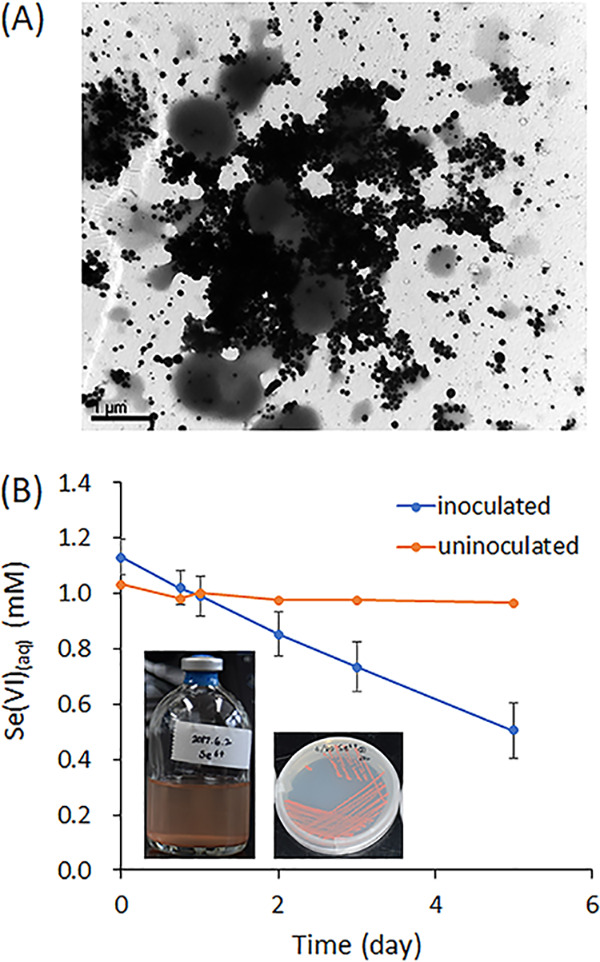
(A) Transmission electron micrograph showing strain SE6-1 with precipitated Se(0). (B) Concentrations of aqueous Se(VI) (SeO_4_^2-^) over time in inoculated and uninoculated incubations of strain SE6-1. Error bars indicate standard deviation values from triplicate incubations.

This study reports the genome of a *Shigella* isolate showing the potential for selenate reduction, which could be of relevance for bioremediation of selenium-contaminated environments. Stream sediment was sampled from a 20-cm depth of a stream bed located in Jeonju, South Korea (35°51′25.6″N, 127°06′00.4″E). One gram of stream sediment was inoculated into 50 ml of 0.1× tryptic soy broth (TSB) amended with 1 mM Na_2_SeO_4_ in a 100-ml serum bottle and purged with 100% nitrogen gas for 30 min. The enrichment was incubated at 28°C for 3 days, and multiple transfer incubations were monitored with the appearance of reddish precipitates as evidence of elemental selenium. From the enrichment serum bottle, aliquots of the solution were serially diluted, spread onto plates of 0.1× tryptic soy agar (TSA) supplemented with 1 mM selenate, and incubated at 28°C in an anaerobic jar with an Oxoid AnaeroGen 2.5-liter sachet. Among dozens of reddish colonies, several strains, including SE6-1, were picked and culture purified by multiple transfer incubations. Genomic DNA was extracted from the cells grown anaerobically in TSA with 1 mM Se(VI) using the G-spin genomic DNA extraction kit (iNtRON Biotechnology, Seongnam, South Korea). The genome of strain SE6-1 was sequenced using a PacBio RS II sequencer (Pacific Biosciences, Menlo Park, CA) and a 20-kb SMRTbell library at ChunLab, Inc. (Seoul, South Korea). *De novo* genome assembly was performed using the Hierarchical Genome Assembly Process (HGAP3) pipeline in single-molecule real-time (SMRT) Analysis v2.3.0 with default parameters. The sequencing generated a total of 83,965 reads with an average length of 9,789 bp. With a genome coverage depth of 167.19×, the assembled genome was 4,762,774 bp in one contig. Gene annotation was performed using the NCBI Prokaryotic Genome Annotation Pipeline (PGAP) v2.0 (1), and 4,440 coding sequences were annotated. A total of 22 rRNA genes and 86 tRNA genes were also identified. To measure overall similarity between genome sequences, orthologous average nucleotide identity (ANI) values were determined using OrthoANI (2). The genome of strain SE6-1 showed the closest alignment with that of Shigella sonnei strain AR_0030 at 98.57% and with that of Escherichia fergusonii strain ATCC 35469 at 89.78%. For the quantitative analysis of selenate reduction, aliquots of medium were withdrawn from triplicate incubations of strain SE6-1 in 0.1× TSB with 1 mM Se(VI), and aqueous selenium concentrations were analyzed using inductively coupled plasma atomic emission spectroscopy (ICP-AES) (ICPS-7500; Shimadzu, Kyoto, Japan) after being filtered with a 0.20-μm syringe filter (Advantec MFS, Inc.) and acidified with 2% HNO_3_. The aqueous selenium represented as selenate was reduced to inorganic elemental selenium as reddish electron-dense precipitates, as indicated by transmission electron microscopy (Fig. 1A), and the concentration decreased over time (Fig. 1B). The strain described will also be useful for better understanding bacterial selenate reduction, especially with the presence of two putative selenate reductase genes, namely, *ygfK* and *ynfE* (3, 4).

### Data availability.

The genome project and sample are indexed in GenBank under BioProject, BioSample, and GenBank accession numbers PRJNA639662, SAMN15244675, and CP055292, respectively. The raw sequencing reads have been deposited in the Sequence Read Archive (SRA) under accession number SRR13487889.
